# The effect of telemedicine in critically ill patients: systematic review and meta-analysis

**DOI:** 10.1186/cc11429

**Published:** 2012-07-18

**Authors:** M Elizabeth Wilcox, Neill KJ Adhikari

**Affiliations:** 1 Department of Medicine, Toronto Western Hospital, and University of Toronto, McLaughlin Wing 2-411H, 399 Bathurst Street, Toronto ON M5T 2S8, Canada; 2 Department of Critical Care Medicine and Sunnybrook Research Institute, Sunnybrook Health Sciences Centre and University of Toronto, 2075 Bayview Avenue Room D1.08, Toronto ON M4N 3M5, Canada

## Abstract

**Introduction:**

Telemedicine extends intensivists' reach to critically ill patients cared for by other physicians. Our objective was to evaluate the impact of telemedicine on patients' outcomes.

**Methods:**

We searched electronic databases through April 2012, bibliographies of included trials, and indexes and conference proceedings in two journals (2001 to 2012). We selected controlled trials or observational studies of critically ill adults or children, examining the effects of telemedicine on mortality. Two authors independently selected studies and extracted data on outcomes (mortality and length of stay in the intensive care unit (ICU) and hospital) and methodologic quality. We used random-effects meta-analytic models unadjusted for case mix or cluster effects and quantified between-study heterogeneity by using *I^2 ^*(the percentage of total variability across studies attributable to heterogeneity rather than to chance).

**Results:**

Of 865 citations, 11 observational studies met selection criteria. Overall quality was moderate (mean score on Newcastle-Ottawa scale, 5.1/9; range, 3 to 9). Meta-analyses showed that telemedicine, compared with standard care, is associated with lower ICU mortality (risk ratio (RR) 0.79; 95% confidence interval (CI), 0.65 to 0.96; nine studies, *n *= 23,526; *I^2 ^*= 70%) and hospital mortality (RR, 0.83; 95% CI, 0.73 to 0.94; nine studies, *n *= 47,943; *I^2 ^*= 72%). Interventions with continuous patient-data monitoring, with or without alerts, reduced ICU mortality (RR, 0.78; 95% CI, 0.64 to 0.95; six studies, *n *= 21,384; *I^2 ^*= 74%) versus those with remote intensivist consultation only (RR, 0.64; 95% CI, 0.20 to 2.07; three studies, *n *= 2,142; *I^2 ^*= 71%), but effects were statistically similar (interaction *P *= 0.74). Effects were also similar in higher (RR, 0.83; 95% CI, 0.68 to 1.02) versus lower (RR, 0.69; 95% CI, 0.40 to 1.19; interaction, *P *= 0.53) quality studies. Reductions in ICU and hospital length of stay were statistically significant (weighted mean difference (telemedicine-control), -0.62 days; 95% CI, -1.21 to -0.04 days and -1.26 days; 95% CI, -2.49 to -0.03 days, respectively; *I^2 ^*> 90% for both).

**Conclusions:**

Telemedicine was associated with lower ICU and hospital mortality among critically ill patients, although effects varied among studies and may be overestimated in nonrandomized designs. The optimal telemedicine technology configuration and dose tailored to ICU organization and case mix remain unclear.

## Introduction

High-intensity physician staffing in intensive care units (ICUs), known as a closed ICU model, is defined as mandatory transfer of responsibility for the care of every critically ill patient to an intensivist-led team or mandatory consultation by an intensivist [1]. This model of staffing is associated with an increased use of evidence-based treatments [2] and significant reductions in mortality and length of stay (LOS) [3,4]. However, projected population demands for intensive care will exceed the number of available intensivists, implying that many critically ill patients will be cared for in low-intensity staffing ICUs (an open model), in which any physician can admit and care for patients without the involvement of an intensivist [5].

Telemedicine, broadly defined as the exchange of medical information via electronic communication, may help to fill gaps in intensivist coverage and give all patients access to specialty care 24 hours per day, 7 days per week [6]. It allows real-time exchange of clinical data and direct interaction among critical care providers across long distances and provides decision support to underserviced rural areas, small hospitals without access to intensivists [7], and large hospitals with low-intensity physician-staffing models or nocturnal physician shortages. Some applications also contain decision-support tools to facilitate implementation of best practices and alarms to alert providers to sudden changes in patient status [8-12].

A recent systematic review found that telemedicine, as compared with standard of care, decreased mortality in patients admitted to ICUs [13]. However, it remains unclear whether an active telemedicine system (for example, one with continuous monitoring of patient data with computer-generated alerts) is required for beneficial clinical effects or whether more-passive systems (such as remote intensivist consultation alone) would suffice. The objective of our systematic review was to determine the effect of telemedicine on ICU mortality in critically ill patients, focusing on subgroup effects related to the intensity of the intervention and quality of the study.

## Materials and methods

### Literature search

OVID versions of MEDLINE (1948 to April, Week 2, 2012), EMBASE Classic and EMBASE (1947 to week 16, 2012); Web of Science (1970 through to April, Week 2, 2012); and the Cochrane Central Register of Controlled Trials (first quarter, 2012) were searched (see Appendix A in Additional file 1). All literature searches were conducted with the aid of an experienced information specialist. We also hand-searched two major intensive care journals, *Critical Care Medicine *and *Intensive Care Medicine *(2001 to 2012); conference abstracts from annual meetings of the Society of Critical Care Medicine and the European Society of Intensive Care Medicine (2001 to 2012); and bibliographies of included studies and personal files. No language restrictions were imposed. Two reviewers independently reviewed all citations; the full text of any citation considered potentially relevant by any reviewer was retrieved. The degree of interrater agreement for study selection was determined by using kappa, with standard definitions for poor (<0.20), fair (0.21 to 0.40), moderate (0.41 to 0.60), good (0.61 to 0.80), and very good (0.81 to 1.00) agreement [14].

### Study selection

Two unblinded reviewers assessed full-text reports and included studies meeting the following criteria: (a) design: randomized and quasi-randomized (allocation by hospital file number, for example) controlled trials or observational studies; (b) population: patients admitted to an ICU; (c) intervention: telemedicine compared with standard of care; and (d) outcome reported: ICU or hospital mortality.

Studies also were considered for inclusion if the telemedicine intervention included cointerventions (for example, computerized physician order entry) and regardless of the degree of exposure to the telemedicine intervention (for example, 24 hours per day, 7 days per week versus nighttime coverage only).

When authors reported data in several publications that included the same patient population, only the most recent or complete study was included in the analysis. Authors were contacted in an attempt to clarify methodology and to request additional data when a study was excluded because the data could not be used [15-21].

### Data abstraction and validity assessment

Two reviewers (MEW, NA) abstracted data including patient population, telecommunication methods, and patient outcomes from included studies. We classified the telemedicine intervention as active (continuous patient data monitoring with computer-generated alerts), high-intensity passive (continuous patient data monitoring without computer-generated alerts), or low-intensity passive (no continuous data monitoring). Study quality was assessed by using the Newcastle-Ottawa score for cohort studies [22].

### Data analysis

The primary outcome of this systematic review was ICU mortality. Secondary outcomes included hospital mortality and ICU and hospital length of stay.

Review Manager 5.0.22 (The Cochrane Collaboration, Oxford, England) was used to calculate pooled risk ratios (RRs) for dichotomous outcomes and pooled weighted mean differences (WMDs) for continuous outcomes, both with 95% confidence intervals (CIs) were calculated. Random-effects models, which incorporate between-trial heterogeneity and thus generally give wider confidence intervals when heterogeneity is present, were used. Because of variability in methods and reporting of adjustment for case-mix and cluster effects among included studies, meta-analyses used unadjusted data. We assessed heterogeneity among trials by using *I^2^*, the percentage of total variability across studies attributable to heterogeneity rather than to chance [23,24] and used published guidelines for low (*I^2 ^*= 25% to 49%), moderate (*I^2 ^*= 50% to 74%), and high heterogeneity (*I^2 ^*≥ 75%) [23]. For the primary outcome of ICU mortality, we inspected a funnel plot (scatterplot of standard error of logRR against RR for each study) and used the Peters regression test [25] to assess for the presence of publication bias. Continuous variables are expressed as mean (standard deviation, SD), unless otherwise indicated.

Subgroup analyses were performed for ICU mortality stratified by (a) study quality (higher, defined as Newcastle-Ottawa score ≥ 6, versus lower), and (b) type of telemedicine intervention: active or high-intensity passive systems versus low-intensity passive systems. In a *post hoc *modification of the second analysis, we compared active with low-intensity passive systems. To test for a subgroup effect, pooled risk ratios (RRs) for each subgroup were compared by using a *z *test [26].

## Results

### Study flow

**Figure 1:**
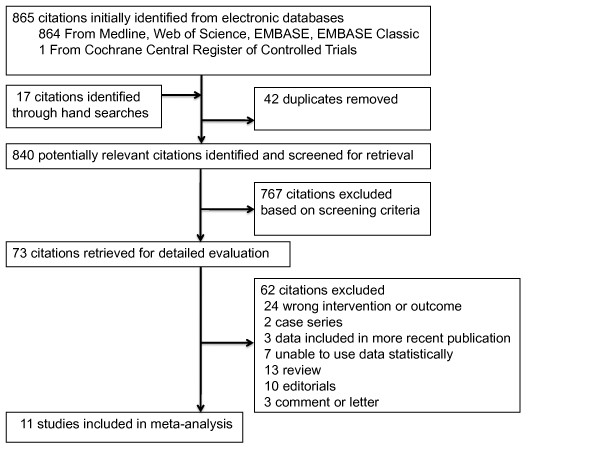
**Flowchart of study selection for the systematic review**.

The search strategy yielded 865 citations (see Figure 1). Seventy-three articles were retrieved for detailed evaluation, of which 62 were excluded (see Appendix B in Additional file 1). Eleven studies (*n *= 49,457) met criteria for inclusion [8-12,27-32]. No authors successfully contacted [16,17,20,21] were able to provide additional data.

### Description of included studies

We did not find any randomized or quasi-randomized trials of telemedicine. Eleven studies used a before-after observational design [8-12,27-32] (Table 1), of which one used a prospective stepped-wedge design [29]. Reviewers achieved very good agreement on study inclusion (kappa 0.92; 95% CI, 0.83 to 1.00). Studies enrolled a median of 2,027 patients (range, 429 to 24,656) and were conducted in one [8,10,11,27-31] or two [12] hospitals, except for two studies that each implemented telemedicine across five hospitals [9,32]. All studies were conducted in the United States. Two studies excluded patients in the ICU for less than 4 hours or transferred from another facility [11,12]. One study included only patients with neurologic diagnoses (stroke, intracranial hemorrhage, traumatic brain injury) [28], another study specifically excluded the neurotrauma ICU from the telemedicine intervention [9], and a third study restricted the intervention to patients with a medical diagnosis [11]. The median study duration in the intervention groups was 37 weeks (range, 10 to 144 weeks) [8-12,27-32].

**Table 1:** Characteristics of the included studies

Source	ICUs/hospitals, *n*	**Patients**^**a **^**(total/pre/post), *n***	Age (years)		Sex (% male)		Illness severity	
			
			Control	Telemedicine	Control	Telemedicine	Control	Telemedicine
Rosenfeld *et al. *2000 [10]	1/1	628/227/201	61	61	56	57	APACHE III 37	APACHE III 38

Breslow *et al. *2004 [8]	2/1	2,140/1,396/744	61	60	56	50	APACHE III APS 39	APACHE III APS 38

Marcin *et al. *2004 [27]	1/1	296/249/47	5.5	5.3	NR	NR	PRISM III 7.5	PRISM III 9.6

Kohl *et al. *2007 [30]	1/1	2,811/189/2,622	NR	NR	NR	NR	NR	NR

Vespa *et al. *2007 [28]	1/1	1,218/578/640	NR	NR	NR	NR	NR	NR

Norman *et al. *2009 [31]	1/1	1,275/356/919	NR	NR	NR	NR	APACHE IV 57	APACHE IV 53

Thomas *et al. *2009 [9]	6/5	4,142/2,034/2,108	60	59	51	53	SAPS II 35	SAPS II 34

McCambridge *et al. *2010 [11]	3/1	1,913/954/959	65	64	50	50	APACHE IV APS 57	APACHE IV APS 58

Morrison *et al. *2010 [12]	4/2	4,088/1,371/2,717	64	65	56	52	APACHE III 49	APACHE III 48

Lilly *et al. *2011 [29]	7/1	6,290/1,529/4,761	62	64	57	57	APACHE III 45	APACHE III 58

Willmitch *et al. *2012 [32]	10/5	24,656/6,504/18,152	NR	NR	NR	NR	CMI 2.68	CMI 2.77

All studies were conducted in the United States. Of the 49,457 patients enrolled, 33,870 were enrolled in the telemedicine arm. Continuous data are expressed as mean (SD). ^a^The number of patients in each category corresponds to the number used in the meta-analysis. APACHE, Acute Physiology and Chronic Health Evaluation; APS, Acute Physiology Score; CMI, Case Mix Index; ICU, intensive care unit; ISS, Injury Severity Score; NR, not reported; PRISM, Pediatric Risk of Mortality Score; SAPS, Simplified Acute Physiology Score.

The median of the mean ages of patients was 62 years (range, 5.5 to 66 years) and 64 years (range, 5.3 to 65 years) in the standard-care and intervention groups, respectively. On average, 54% of patients were men (range, 51% to 57%) in the six studies providing this information [8,10-12,29]. Patients had high illness severity, measured by using a variety of scoring systems (Table 1).

### Details of the interventions

Three studies implemented a low-intensity passive system [10,27,28] (Table 2). One study implemented a low-intensity passive system with video teleconferencing equipment exclusively for clinical consultation [27]. On average, 1.5 consults occurred per patient (range, one to seven) during the ICU stay [27]. Another study used a low-intensity passive system featuring a robotic telepresence program and a clinical information system as a cointervention [28]. In this study, the tele-ICU physician assessed patients twice daily and reviewed each patient during nighttime rounds (minimum of 5 minutes per patient; mean, 52 minutes per night). The third study used video-conferencing equipment to perform formal rounds with either the attending physician or senior housestaff [10]. Tele-ICU physicians devoted 4 to 5 hours per day to patient care; physiological data from patients were stored and viewed every 2 hours.

**Table 2:** Telemedicine intervention in the included studies

Source Study periods	Type of hospital/ICU ICU staffing model **Type of intervention**^**a**^	Intervention details	Intervention dose	Equipment cointerventions
Rosenfeld *et al. *2000 [10] Pre 1: 1 Sept-18 Dec 1996 Pre 2: 1 Feb-18 May 1997^b^ Post: 1 Sept-18 Dec 1997	Academic-affiliated community hospital; surgical ICU Open model^c^ Low-intensity passive	Tele-intensivist interacted with patients and healthcare personnel via dedicated video conferencing and data transmission equipment 24 hours/day Clinical and stored physiologic data reviewed q2hours	Formal video conferencing rounds occurred on 50% of days; otherwise, intensivist discussed each case with senior housestaff or attending physician Tele-intensivists spent 4 to 5 hours/day on clinical care	Spacelabs Medical, Seattle WA None

Breslow *et al. *2004 [8] Pre: 1 July 1999-20 June 2001 Post: 1 Jan-30 June 2001	Tertiary care, teaching; medical and surgical ICUs Closed unit for teaching team of medical ICU patients (40%); open model for remaining medical ICU patients and surgical ICU^c^ High-intensity passive or active (alerts not clearly described)	Tele-ICU staff (board certified intensivist, nurse) monitored all patients 19 hours/day (1200-0700) Admitting physician determined tele-ICU decision-making authority (all versus some versus off-hours) Tele-ICU reviewed patient data q4hours	Not described	VISICU Inc. (eICU CARE), Baltimore MD None

Marcin *et al. *2004 [27] Pre: Oct 1997-Sept 1998 Post: Apr 2000-Apr 2002^d^	Tertiary referral; adult ICU (with some pediatric patients) Pediatric intensivist during baseline period only Low-intensity passive	Consultation (at discretion of admitting physician) with tele-pediatric intensivist using portable telemedicine unit in pediatric ICU and five consultants' homes available 24 hours/day within 15 minutes	Number of consultations, one to seven per patient (median, 1; mean, 1.5)	Tandberg 800 video conference units None

Kohl *et al. *2007 [30] Dates not reported	Academic; surgical ICU Staffing model not described High-intensity passive or active (based on vendor)	Tele-ICU staffed by board certified intensivists; no further details provided	Not described	VISICU Inc. (eICU CARE), Baltimore MD None

Vespa *et al. *2007 [28] Pre: 2003-2004 fiscal year Post: June 2005-June 2006	Academic; neurologic ICU Staffing model not described; tele-intensivist same as on-site intensivist Low-intensity passive	Robotic telepresence program for live interactive consultation and review of physiologic trends with intensivist [2000-0000 (weekdays); 1800 (weekends)] Each patient reviewed for ≥ 5 minutes	Mean, two sessions/day Mean night-time rounding session, 52 minutes	Robot: InTouch Health, Santa Barbara CA Informatics system: Global Care Quest, Aliso Viejo CA Integrated clinical information system Paging protocol with goal of attending physician response within 15 minutes

Norman *et al. *2009 [31] Pre: Jan-Mar 2008 Post: Jan-Mar and Apr-June 2009^e^	Hospital not described; medical-surgical ICU Staffing model not described High-intensity passive or active (alerts not clearly described)	Tele-ICU staff ("team" included nurse; intensivist presence not specifically stated) reviewed patients; no further details provided	Not described	VISICU Inc. (eICU CARE), Baltimore MD Electronic discharge management tool

Thomas *et al. *2009 [9] Pre: Jan 2003-Aug 2005 Post (staggered roll-out): July 2004-July 2006	Closed^f ^medical and trauma/surgical ICU in tertiary care teaching hospital; two open medical-surgical ICUs in two small community hospitals; two open medical-surgical ICUs in two large urban hospitals Active	Tele-ICU staffed by two physicians (noon -7 am Monday-Friday, 24 hours/day weekends), four registered nurses, and two administrative technicians Rounds frequency: severely ill q1 hour, moderately ill q2 hours, relatively stable q4 hours Local physicians delegated to tele-ICU authority for full treatment (31% of patients) or for intervention only for life-threatening events (66%)	Tele-ICU physicians gave 1,446 orders in 60 days (four ICUs) Two closed ICUs, 5.3 orders/day (7% high-level interventions, (for example, code supervision, ventilator management) Two open ICUs, 18.5 orders/day (26% high-level)	VISICU Inc. (eICU CARE), Baltimore MD None

McCambridge *et al. *2010 [11] Pre: Sept 2002-Dec 2003 Post: Oct 2004-July 2005	Academic community hospital; three ICUs Closed model^f^ Active	Tele-ICU team (intensivist and critical care nurse) (1900-0700) admitted new patients and responded to phone calls from ICU nurses, computer-generated alerts, and radiographic abnormalities Rounds for all monitored patients q2 hours	Not described	Vistacom Inc, Allentown PA Health information technology bundle: EMR with automatic alerts (*i*MD*soft*, Needham MA); CPOE, electronic MAR and bar-coded medication administration, PACS (GE Healthcare, Fairfield CT)

Morrison *et al. *2010 [12] Pre: Dec 2002-Mar 2003 Post 1: Dec 2004-Mar 2004 Post 2: July-Oct 2004^g^	One community teaching hospital (medical ICU, surgical ICU, cardiac ICU) and one community nonteaching hospital (medical-surgical ICU) Open model^c^ Active	Admitting physician responsible for care plan and determined involvement of tele-ICU (four categories from emergency care only to no restrictions) Tele-intensivist reviewed all patient data at least q4 hours (q1 hour for sickest patients) At teaching hospital, tele-intensivist supervised and taught housestaff "real-time"	Physician adoption of high-level (unrestricted) tele-ICU care differed (teaching hospital, 25% of physicians [post one], 57% [post two]; nonteaching hospital, 9% [post one], 27% [post two])	VISICU Inc. (eICU CARE), Baltimore MD, including "Sentry Alerts" software

Lilly *et al. *2011 [29] Pre: April 2005-Feb 2007 Post: (staggered roll-out) Aug 2006-Sept 2007	Academic medical center; seven ICUs: three medical, three surgical, and one mixed cardiovascular Closed model^f^ Active	Tele-ICU (hospital staff intensivist, affiliate practitioner, systems analyst, ≥ one data clerks), 24 hours/day Tele-ICU monitored 5-minute timed median vital sign values on electronic flow sheet; reviewed care; audited best-practice adherence real-time; reviewed night-time admissions; monitored electronic alerts, intervened when responses of bedside clinicians to in-room alarms delayed	Tele-ICU reviewed care plan for 48% of after-hours admissions (46% reviewed by other methods in pre period) 23 943 tele-ICU initiated interventions for physiologic instability that affected care plan (76% "major")	VISICU Inc. (eICU CARE), Baltimore MD; APACHE (Cerner Healthcare Solutions, Kansas City MO) Criticalware (UMass) software package to audit best practices (glycemic control; prevention of DVT, CRBSI, VAP) None

Willmitch *et al. *2012 [32] Staggered roll-out: Dec 2005-July 2007 Pre: 1 year before roll-out Post 1: year 1 after roll-out Post 2: year 2 after roll-out Post 3: year 3 after roll-out^h^	Five community hospitals with 10 ICUs Closed model^f ^in largest hospital (28% of ICU beds in the study); otherwise open model^c^ Active	Tele-ICU, staffed by one intensivist, three critical care nurses, and one secretary, 24 hours/day	All admitting and consulting physicians (*n *= 2,607) indicated level of tele-ICU intervention for their patients: 1% selected level I (emergency care only), 97% level II (best-practices adjustments), 2% level III (no restrictions)	Philips VISICU eCare Manager (Admission, discharge and transfer interfaces), Philips Smart Alerts, Philips VISICU camera system (Philips, Amsterdam, Netherlands) None

CPOE, computerized physician order entry; CRBSI, catheter-related bloodstream infection; DVT, deep vein thrombosis; EMR, electronic medical record; ICU, intensive care unit; MAR, medication administration record; PACS, picture archiving and communications system; VAP, ventilator-associated pneumonia. ^a^Active system: continuous data monitoring with computer-generated alerts; high-intensity passive: continuous data monitoring without computer-generated alerts; low-intensity passive: no continuous data monitoring. ^b^We used data from both pre (baseline) periods. ^c^Open model refers to low-intensity on-site daytime intensivist staffing, in which patients may be cared for in the ICU without the mandatory involvement of an intensivist in their care. ^d^Patients assigned to the control group in the meta-analysis include those from the baseline period and concurrent controls from the intervention period who did not receive a telemedicine consultation. ^e^Patients in both intervention periods were included in the meta-analysis. ^f^Closed model refers to high-intensity on-site daytime intensivist staffing, in which an intensivist must primarily manage or consult on all patients admitted to the ICU. ^g^We used data from both post (intervention) periods. ^h^We used data from all post (intervention) periods.

Five studies examined active interventions with audio-video monitoring and a data-monitoring system that generated alerts based on abnormal vital signs or laboratory or radiologic tests [9,11,12,29,32], and two studies did not provide enough detail to classify them as either high-intensity passive or active systems [8,30,31]. Six studies used VISICU software (eICU program of intensivist-led remote monitoring; VISICU Inc., Baltimore, MD, USA, and Amsterdam, The Netherlands) [8,9,12,29,31,32]. Patients were monitored continuously for 12 to 24 hours per day, with tele-ICU physicians rounding on all monitored patients every 1 to 4 hours. Studies provided varying levels of detail on the dose of the intervention; three studies reported on actual patient-care time [10,28] or number of consultations provided [27], two studies described the number and nature of patient-care orders given by the tele-ICU [9,29], and two studies described different levels of tele-ICU involvement in patient care, depending on hospital physician preference in primarily open ICUs [12,32]. One study measured changes in adoption of best practices with telemedicine [29], whereas another reported that the tele-ICU had the authority to make recommendations regarding best practices for some patients, but did not report process-of-care data [32].

### Study quality

Overall study quality was moderate (mean score on Newcastle-Ottawa scale, 5.1; range, 3 to 9 (maximum possible score); see Appendix C in Additional file 1). A minority of studies adequately reported details of the uptake of the intervention [9,10,27-29] and assessment of outcome [10,29].

### Primary and secondary outcomes

**Figure 2:**
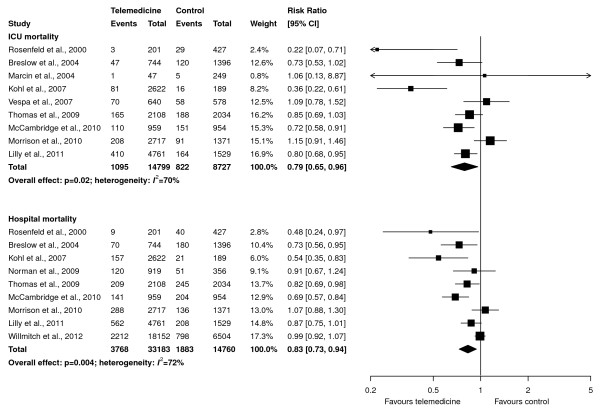
**Effect of telemedicine on ICU mortality (upper panel) and hospital mortality (lower panel)**. The pooled risk ratio with 95% confidence interval (CI) was calculated by using a random-effects model. Weight refers to the contribution of each study to the overall estimate of treatment effect.

Pooled data from nine studies (23,526 patients, 14,799 in the telemedicine group) showed that telemedicine reduced ICU mortality (RR, 0.79; 95% CI, 0.65 to 0.96; *P *= 0.02; Figure 2, upper panel). Visual inspection of a funnel plot and Peters regression test (*P *= 0.45) did not suggest publication bias (see Appendix D in Additional file 1). Similarly, data from nine studies (47,943 patients, 33,183 in the telemedicine group) showed that telemedicine reduced hospital mortality (RR, 0.83; 95% CI, 0.73 to 0.94; *P *= 0.004; Figure 2, lower panel). Moderate statistical heterogeneity was found in both analyses (*I^2^*= 70% and 72%, respectively).

**Figure 3:**
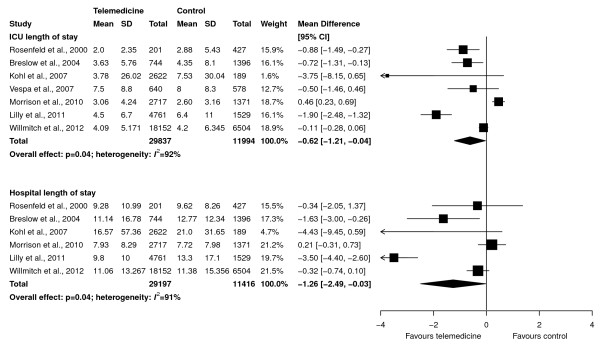
**Effect of telemedicine on ICU length of stay (upper panel) and hospital length of stay (lower panel) in days**. The pooled weighted mean difference with 95% confidence interval (CI) was calculated by using a random-effects model. Weight refers to the contribution of each study to the overall estimate of treatment effect.

Pooled data from seven studies (41,831 patients, 29,837 in the telemedicine group) showed a statistically significant difference in ICU length of stay (WMD, -0.62 days; 95% CI, -1.21 to -0.04 days; *P *= 0.04; Figure 3, upper panel). Similarly, a statistically significant difference was found in hospital length of stay (WMD, -1.26 days; 95% CI, -2.49 to -0.03 days; *P *= 0.04; Figure 3, lower panel) in six studies (40,613 patients, 29,197 in the telemedicine group). Both analyses showed high between-study heterogeneity (*I^2 ^*>90%).

### Subgroup analyses

**Figure 4:**
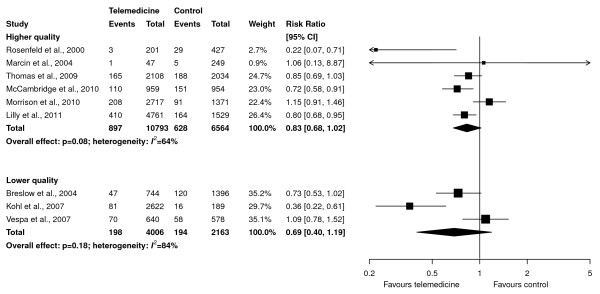
**Subgroup analyses of effect of telemedicine on ICU mortality based on study quality (higher quality in upper panel and lower quality in lower panel)**. Pooled risk ratios were calculated by using a random-effects model. Weight refers to the contribution of each study to each subgroup's estimate of treatment effect. The interaction *P *value for the difference between risk ratios is 0.53.

The effect of telemedicine on ICU mortality was similar in higher-quality studies (RR, 0.83; 95% CI, 0.68 to 1.02; *P *= 0.08; six studies, 17,357 patients, 10,793 in the telemedicine group; Figure 4, upper panel) and lower-quality studies (RR, 0.69; 95% CI, 0.40 to 1.19; *P *= 0.18; three studies, 6,169 patients, 4,006 in the telemedicine group; Figure 4, lower panel). These RRs were not statistically different (*P *= 0.53 for test for interaction).

**Figure 5:**
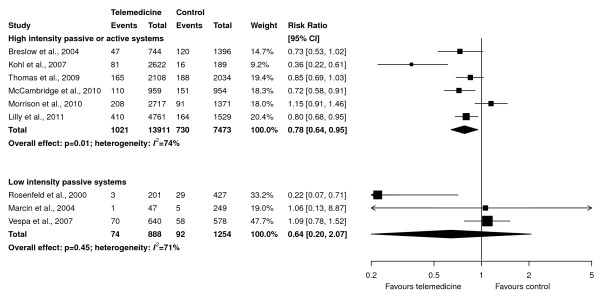
**Subgroup analyses of effect of telemedicine on ICU mortality based on intensity of the intervention (continuous patient-data monitoring, with or without computer-generated alerts (active or high-intensity passive systems), in upper panel, and neither present (low-intensity passive systems) in lower panel)**. Pooled risk ratios were calculated by using a random-effects model. Weight refers to the contribution of each study to each subgroup's estimate of treatment effect. The interaction p value for the difference between risk ratios is 0.74.

Active or high-intensity passive telemedicine interventions (continuous data monitoring with or without computer-generated alerts) significantly decreased ICU mortality (RR, 0.78; 95% CI, 0.64 to 0.95; *P *= 0.01; six studies, 21,384 patients, 13,911 in the telemedicine group; Figure 5, upper panel), compared with studies with passive interventions (remote intensivist consultation) (RR, 0.64; 95% CI, 0.20 to 2.07; *P *= 0.45; three studies, 2,142 patients, 888 in the telemedicine group; Figure 5, lower panel). However, these RRs were not statistically different (*P *= 0.74 for a test of interaction). When only active interventions were considered, no effect on ICU mortality was found (RR, 0.86; 95% CI, 0.72 to 1.03; *P *= 0.10; four studies, 16,433 patients, 10,545 in the telemedicine group), and no difference from the effect in the passive group was found (*P *= 0.62 for test of interaction).

## Discussion

In this systematic review and meta-analysis, we found 11 before/after observational studies, including 49,457 patients, that examined the effect of telemedicine on clinically important outcomes. Our main findings are that telemedicine reduced ICU and hospital mortality and lengths of stay in critically ill patients. Although the effect of telemedicine on ICU mortality was similar among active and passive systems, this subgroup analysis was underpowered to detect a true dose-response effect. Notwithstanding the methodologic limitations of observational studies and meta-analyses of unadjusted data, telemedicine appears to be a promising intervention to improve outcomes in the ICU and should be studied further.

Telemedicine systems may improve clinical outcomes by improving adherence to best practices in the ICU, which has been demonstrated to be poor [33]. One study [29] in this review found that telemedicine increased adherence to four best practices and reduced complications of ventilator-associated pneumonia and catheter-related bloodstream infections. Other studies not meeting inclusion criteria for this review have also shown improved adherence to best practices with telemedicine. For example, a recent cluster-randomized trial showed that a multifaceted knowledge-translation intervention that included education via telemedicine increased the adoption of targeted best practices in community hospitals with few resources dedicated to quality improvement [34]. Similarly, a before/after study found a significant increase in the percentage of patients who received a daily sedative interruption with tele-ICU pharmacy support [35]. Last, one study reported improved nursing-staff satisfaction after the implementation of robotic tele-ICU [36]. However, with this exception [29], the studies in this review did not report process-of-care measures sufficiently to address the hypothesis that lower mortality was associated with improved adherence to best practices.

Our results are similar to those of Young *et al. *[13], who found that telemedicine is associated with a reduction in ICU mortality (pooled odds ratio, 0.80; 95% CI, 0.66 to 0.97; *P *= 0.02), but not hospital mortality. Whereas Young *et al. *explored subgroup effects on the basis of vendor affiliation, our objective was to determine whether more technologically advanced telemedicine systems, with or without continuous patient-data monitoring, were associated with a statistically significant benefit on ICU mortality compared with less-advanced systems. Our subgroup analysis did not confirm a differential effect between these types of systems, even when the advanced-technology group was restricted to active systems with continuous patient-data monitoring and alerts. In addition to lack of statistical power in this analysis, the interventions used in included studies may have been misclassified because of the lack of a commonly adopted conceptual framework and vocabulary to describe components of the intervention, the intensity with which they were deployed, and the clinical actions attributable to their deployment [37].

Besides the lack of evidence to guide selection of telemedicine features important for clinical benefit, other issues should mitigate against widespread implementation. First, generalizability is limited, as studies were conducted in few hospitals in one country. Second, the impact of telemedicine likely depends on characteristics of the environment in which it is deployed, including ICU organization (such as physician-staffing model and use of protocols) and patient case mix. Studies generally reported limited relevant contextual details. Third, the costs of installation and maintenance [38], potential for malfunction and downtime, and impact of redeployment of intensivists away from bedside clinical care during labor shortages have not been analyzed. Fourth, telemedicine has been primarily examined in before/after observational studies, from which conclusions regarding causality may be confounded by secular trends in case-mix and other interventions. These issues, although shared by healthcare technology in general [39], imply that universal adoption of telemedicine should be depend on the results of future studies, the design of which should be informed by a robust understanding of system design and organizational factors associated with patient benefit.

Strengths of this review include several methods to minimize bias, including a comprehensive literature search, duplicate outcomes abstraction, consideration of important clinical outcomes, and use of an established method to assess study quality specific to nonrandomized studies [22]. Our review also has weaknesses. In the absence of any randomized trials of telemedicine, we included observational studies, which tend to overestimate the effects of an intervention [40] even with standard methods to adjust for differences between groups [40-42]. Our meta-analyses used unadjusted data and may have further exaggerated treatment effects. Although we believed the interventions were sufficiently similar in concept and execution to permit statistical aggregation, major differences occur in the components of this intervention, methods of deployment, and rates of adoption in the included studies. Moderate statistical heterogeneity was seen in our primary outcome that persisted in subgroups defined by study quality and telemedicine technology. Therefore, even among studies using advanced telemedicine interventions, our review cannot identify with certainty the components essential to success. Last, our subgroup analyses had few studies; for example, the lower-technology (low-intensity passive) telemedicine subgroup had only three studies. The power to detect clinically important subgroup effects was therefore limited.

## Conclusions

Telemedicine is a promising technology to reduce mortality in the critically ill. Recently, the Critical Care Societies Collaborative proposed a comprehensive research agenda in ICU telemedicine, including development of a conceptual framework to describe the telemedicine system and the recipient ICUs and elucidation of mechanisms of telemedicine's effects on downstream clinical outcomes by analyzing effects on structure and process-of-care variables [37]. We believe that this complex intervention, similar to others in the ICU, warrants eventual evaluation in a cluster-randomized trial. Important areas of research that would inform the design of such a trial, in addition to those described, include individual patient meta-analysis of existing studies to permit adjustment for hospital and patient-level characteristics to identify patients and centers most likely to benefit, assessment of clinical equipoise (physician and nursing attitudes to ICU telemedicine) by using qualitative methods [43], and pilot observational studies to establish the optimal telemedicine technology configuration and dose tailored to ICU organization and case mix.

## Key messages

• We found 11 before/after observational studies including 49,457 patients that examined the effect of telemedicine on clinically important outcomes.

• Pooled unadjusted data from nine studies showed that telemedicine reduced ICU mortality (RR, 0.79; 95% CI, 0.65 to 0.96; *P *= 0.02) and hospital mortality (RR, 0.83; 95% CI, 0.73 to 0.94; *P *= 0.004); reductions in ICU and hospital lengths of stay were also statistically significant.

• The effect of telemedicine on ICU mortality was similar in active or high-intensity passive systems (continuous patient-data monitoring with or without electronic alerts) compared with low-intensity passive systems (remote intensivist consultation only), but this subgroup analysis was underpowered.

• Future research should establish the optimal telemedicine technology configuration and dose tailored to ICU organization and case mix.

## Abbreviations

CI: confidence interval; ICU: intensive care unit; LOS: length of stay; RR: risk ratio; WMD: weighted mean difference.

## Competing interests

The authors declare that they have no competing interests.

## Authors' contributions

MEW conceived of the study, searched the literature, selected studies for inclusion, abstracted data, analyzed data, wrote the first draft of the manuscript, and revised the manuscript. NKJA selected studies for inclusion, abstracted data, analyzed data, and revised the manuscript. Both authors approved the final manuscript.

## Supplementary Material

Additional file 1 **Supplementary details of method and results**. This file contains details of the search strategy (Appendix 1), summary of reasons for excluding 62 studies (Appendix B), assessment of methodologic quality of the included studies (Appendix C), and a funnel plot for the meta-analysis of ICU mortality (Appendix D). Click here for file
