# Supplementary material for: The effect of telemedicine in critically ill patients: systematic review and meta-analysis
Source: Crit Care. 2012 Jul 18;16(4):R127. doi: 10.1186/cc11429 (PMC3580710; doi:10.1186/cc11429)
Supplement: Additional file 1 — Supplementary details of method and results. This file contains details of the search strategy (Appendix 1), summary of reasons for excluding 62 studies (Appendix B), assessment of methodologic quality of the included studies (Appendix C), and a funnel plot for the meta-analysis of ICU mortality (Appendix D). [file cc11429-S1.DOC]

**Appendix A. Search strategy**

OVID versions of MEDLINE (1948 through to April, Week 2 2012), EMBASE Classic and EMBASE (1947 through to Week 16, 2012); Web of Science (1970 through to April, Week 2 2012); and the Cochrane Central Register of Controlled Trials (first quarter, 2012) [telemedicine* and critical care] were searched. The strategy for MEDLINE, EMBACS Classic, and EMBASE was as follows:

1. Exp intensive care units/ or intensive care unit$.mp.
2. Exp critical care/ or critical care.mp.
3. 1 or 2
4. Exp mortality/ or mortality.mp.
5. Exp hospitali#ation/ or hospitali#ation.mp.
6. Exp length of stay/ or length of stay.mp. or LOS.mp.
7. 4 or 5 or 6
8. Exp telemedicine/ or telemedicine.mp.
9. eICU.mp.
10. tele-ICU.mp.
11. teleintensivist.mp.
12. 8 or 9 or 10 or 11
13. 3 and 7 and 12
14. remove duplicates from 13

Notes: ‘$’ retrieves unlimited suffix variations; the .mp. extension includes the title, original title, abstract, and subject heading fields in all databases; ‘#’ retrieves all variations of spelling of all textwords whenever relevant (i.e. British spelling of hospitalization in item 5).

**Appendix B. Summary of reasons for exclusion of 62 studies from systematic review**

**Intervention or outcome not of interest (n=24)**

Chung K, et al. Robotic telepresence in a military ICU: A prospective observational study [abstract]. Crit Care Med 2009; 37(12): A900.

Coletti C, Elliott D, Zubrow M. Resident perceptions of an integrated remote ICU monitoring system [abstract]. Crit Care Med 2008; 36(12): A290.

Franzini L, Thomas E. Costs and effectiveness of tele-ICUs in reducing morbidity and mortality in intensive care units. J Med Econ 2008;11(1): 165-169.

Grundy BL. Telemedicine in Critical Care - Preliminary report. Crit Care Med 1976; 4(2): 104-105.

Grundy BL, Jones P, Lovitt A. Telemedicine in Critical Care - Analysis of an 18 month experience. Crit Care Med 1978; 6(20):1 26.

Grundy BL, et al. Telemedicine in critical care: an experiment in health care delivery. JACEP 1977; 6(10): 439-44.

Grundy BL, Jones PK, Lovitt A. Telemedicine in Critical Care - Problems in design, implementation, and assessment. Crit Care Med 1982; 10(7): 471-475.

Heath BW, et al. Pediatric critical care telemedicine in a rural underserviced area. Crit Care Med 2007; 35(12): 1024.

Heath BW, et al. Pediatric critical care telemedicine in rural underserviced emergency departments. Pediatr Crit Care Med 2009; 10(5): 588-591.

Hitt JA, et al. The economic and clinical value of a remote intensive care unit [abstract]. Crit Care Med 2007; 35(12): A106.

Jenkins C. Improved screening and management of severe sepsis (SS): Combining an integrated multidisciplinary team and technology [abstract]. Crit Care Med 2009; 37(12): A738.

Marttos A, et al. Telerounds in a trauma ICU dept [abstract]. Crit Care Med 2008; 36(12): A225.

McNelis J, et al. Off hours remote presence technology (RPT) enhances communication between intensivists and staff [abstract]. Crit Care Med 2008; 36(12): A106.

Rendina MC. The effect of telemedicine on neonatal intensive care unit length of stay in very low birthweight infants. Proc AMIA Symp: 111-5.

Rendina MC, et al. Effect of telemedicine on health outcomes in 87 infants requiring neonatal intensive care. Telemed J 1998; 4(4): 345-51.

Rincon T, Seiver A, Farrell W, et al. Increased documentation of ICD-9-CM codes 995.92 and 785.52 with template-oriented monitoring and screening by a tele-ICU. Crit Care Med 2009;37(12): A8.

Salerno RA, et al. Pediatric critical care telemedicine in a rural underserviced area: the transport team perspective [abstract]. Crit Care Med 2007; 35(12): A100.

Thomas C, Prasanna M. The role of a 'satellite-service' in the national organisation of burn care in the Sultanate of Oman. Burns 2000; 26(2): 181-5.

Thomas EJ, et al. The impact of a tele-ICU on provider attitudes about teamwork and safety climate [abstract]. Crit Care Med 2007; 35(12): A529.

Westbrook JI, et al. Impact of an ultrabroadband emergency department telemedicine system on the care of acutely ill patients and clinicians' work. Med J Aust 2008; 188(12): 704-8.

Wheeler T. Communications special need profiles. Telemed Today 1998; 6(4): 21.

Yager P, et al. Use of telemedicine to provide enhanced communication between at-home attendings and bedside personnel in a pediatric intensive care unit [abstract]. Crit Care Med 2010; 38(12): A105.

Zawada ET, et al., Clinical and fiscal impact of rural tele-intensivist staffing program on transfer of patients from their community to tertiary care hospital [abstract]. Crit Care Med 2008; 36(12): A350.

Zawada ET, Herr P. ICU telemedicine improves care to rural hospital reducing costly transports [abstract]. Crit Care Med 2008; 36(12): A668.

**Case series (n=2)**

Berg BW, Vincent DS, Hudson DA. Remote critical care consultation: telehealth projection of clinical specialty expertise. J Telemed Telecare 2003; 9: S9-S11.

Kon AA, Marcin JP. Using telemedicine to improve communication during paediatric resuscitations. J Telemed Telecare 2005; 11(5):261-4.

**Findings incorporated in a more recent publication (n=3)**

Marcin JP, et al. The use of telemedicine to provide pediatric critical care consultations to pediatric trauma patients admitted to a remote trauma intensive care unit: a preliminary report. Pediatr Crit Care Med 2004; 5(3):251-6.

Thomas EJ, et al. Impact of a tele-ICU on mortality, complications, and length of stay in six ICUs [abstract]. Crit Care Med 2007; 35(12): A30.

Zawada ET, et al. Prognostic outcomes after the initiation of an electronic telemedicine intensive care unit (eICU) in a rural health system. S D Med 2006; 59(9): 391-3.

**Unable to use data statistically (n=7)**

Chen J, et al. The clinical application of remote critical care network. Zhongguo Wei Zhong Bing Ji Jiu Yi Xue 2009; 21(11): 679-681.

Dimand RJ, et al. Use of telemedicine to provide pediatric critical care inpatient consultations to underserved rural Northern California [abstract]. Crit Care Med 2001; 29(12 Suppl): A112.

Rogove H, et al. Enhanced access to neurointensivists through a telemedicine program. Crit Care Med 2009; 37(12): A2.

Turner G, et al. Predictable changes in medical-surgical ICU patient hospital mortality with evolving intensivist services and evolving then devolving telemedicine unit services [abstract]. Crit Care Med 2009; 37(12): A130.

Latif A, et al. A consultative telemedicine service improves compliance with best practice guidelines in a highly staffed intensive care unit [abstract]. Crit Care Med 2010; 38(12): A582.

Marini CP, et al. The effect of telerounding in a surgical intensive care unit. Critical Care Medicine 2007;35(12):[abstract] A627.

Zawada ET, et al. Impact of an intensive care unit telemedicine program on a rural health care system. Postgraduate Medicine 2009;121(3):160-170.

**Review (n=13)**

Boots et al. Remote care by telemedicine in the ICU: Many models of care can be effective. Current Opinion in Critical Care 2011; 17(6): 634-640.

Breslow MJ. ICU telemedicine. Organization and communication. Crit Care Clin 2000; 16(4): 707-22.

Breslow MJ. Remote ICU care programs: Current status. J Crit Care 2007; 22(1): 66-76.

Cregan P, et al. The ViCCU Project - Achieving Virtual Presence using Ultrabroadband INternet in a Critical Clinical Application - Initial Results. Medicine Meets Vitual Reality 13: The Magic Next Becomes the Medical Now 2005; 111: 94-98.

Gamble C. Remote control. Specialists are running intensive-care units from remote sites via computers, and at least one health system with the eICU is reaping financial rewards--and saving lives. Mod Healthc 2002; 32(8): 40-2.

Groves RH, Holcomb BW, Smith ML. Intensive care telemedicine: evaluating a model for proactive remote monitoring and intervention in the critical care setting. Stud Health Technol Inform 2008; 131: 131-46.

Hoffmann S. Counterpoint: Should tele-ICU services be eligible for professional fee billing? No. Chest 2011; 140(4).

Hulshoff L et al. Telemedicine in the ICU, a review. Netherlands Journal of Critical Care 2011; 15(1): 9-12.

McCambridge MM, Tracy JA, Sample GA. Point: Should tele-ICU services be eligible for professional fee billing? Yes. Tele-ICUs and the triple aim. Chest 2011; 140(4).

Murias G, et al. Telemedicine: Improving the quality of care for critical patients from the pre-hospital phase to the intensive care unit. Med Intensiva 2010; 34(1): 46-55.

Nguyen Y-L, Kahn JM, Angus DC. Reorganizing adult critical care delivery: the role of regionalization, telemedicine, and community outreach. Am J Respir Crit Care Med 2010; 181(11): 1164-9.

Rosenfeld B. A remote possibility. Cost Qual 2000; 6(4): 38-9.

Rosenfeld B. eICU: More data are now available. Health Aff (Millwood) 2009; 28: 6.

**Editorial (n=10)**

Barro S, et al. Intelligent telemonitoring of critical-care patients. IEEE Eng Med Biol Mag 1999; 18(4): 80-88.

Becker C, Remote control. Specialists are running intensive-care units from remote sites via computers, and at least one health system with the e-ICU is reaping financial rewards--and saving lives. Mod Healthc 2002; 32(8): 40-2.

Gamble KH. Critical care network. ICU telemedicine can help ease the burden of caring for critically ill patients--provided all the right pieces are in place. Healthc Inform 2009; 26(12): 26.

Kahn JM, Hall JB. More doctors to the rescue in the intensive care unit: A cautionary note. Am J Respir Crit Care Med 2010; 181(11):1160-1161.

Kahn JM. Intensive care unit telemedicine: Promises and pitfalls. Arch Intern Med 2011; 171(6):495-496.

Kahn JM. The use and misuse of ICU telemedicine. JAMA 2011; 305(21):2227-8.

Kozar RA, Shackford SR and Cocanour CS. Challenges to the care of the critically ill: Novel staffing paradigms. J Trauma 2008; 64(2): 366-370.

Leong JR, Sirio CA, Rotondi AJ. eICU program favorably affects clinical and economic outcomes. Crit Care 2005; 9(5):E22.

Lustbader D, Fein A. Emerging trends in ICU management and staffing. Crit Care Clin 2000; 16(4):735.

Shafazand S, Shigemitsu H, Weinacker AB. A brave new world: remote intensive care unit for the 21st century. Crit Care Med 2000; 28(12): 3945-6.

**Comment or letter (n=3)**

Davis TM, Jackson WL. Specific elements of teleintensivist paradigm require additional scrutiny and justification. Arch Intern Mede 2010; 170(16): 1509-10; author reply 1510.

McCambridge M, et al. In reply. Arch of Intern Med 2010; 170(16):13.

Metske HA, Spronk PE, Schultz MJ. Evaluating tele-ICU reengineering of critical care processes. JAMA 2011; 306(13):1441.

**Appendix C: Assessment of methodologic quality of included studies**

| **Source** | **Selection (4)** | **Comparability (2)** | **Outcomes (3)** | **Total (9)** |
| --- | --- | --- | --- | --- |
| Rosenfeld et al., 2000 | 4 | 2 | 3 | 9 |
| Breslow et al., 2004 | 3 | 1 | 1 | 5 |
| Marcin et al., 2004 | 4 | 1 | 2 | 7 |
| Kohl et al., 2007 (30) | 1 | 0 | 2 | 3 |
| Vespa et al., 2007 | 2 | 0 | 1 | 3 |
| Norman et al., 2009 (31) | 2 | 0 | 2 | 4 |
| Thomas et al., 2009 | 4 | 2 | 2 | 8 |
| McCambridge et al., 2010 | 3 | 1 | 2 | 6 |
| Morrison et al., 2010 | 3 | 2 | 2 | 7 |
| Lilly et al., 2011 (29) | 3 | 2 | 3 | 8 |
| Willmitch et al., 2012 (32) | 1 | 2 | 2 | 5 |

Study quality was assessed using the Newcastle-Ottawa Score . The number in parentheses after the headings of columns 2-5 refers to the maximum possible score. Points for selection are given for representativeness of the exposed cohort, selection of the non-exposed cohort, ascertainment of exposure, and demonstration that outcome of interest was not present at start of study. Points for comparability are given for comparability of cohorts on the basis of the design or analysis. Points for outcomes are given for assessment of outcome, adequacy of length of follow-up, and adequacy of follow-up.

See main text for reference numbers.

**Appendix D. Funnel plot for pooled analysis of ICU mortality. Each point represents one trial. RR, risk ratio; SE, standard error.** There was no evidence of publication bias (p=0.45) using Peters’ regression test (JAMA 2006;295:676-680).

**
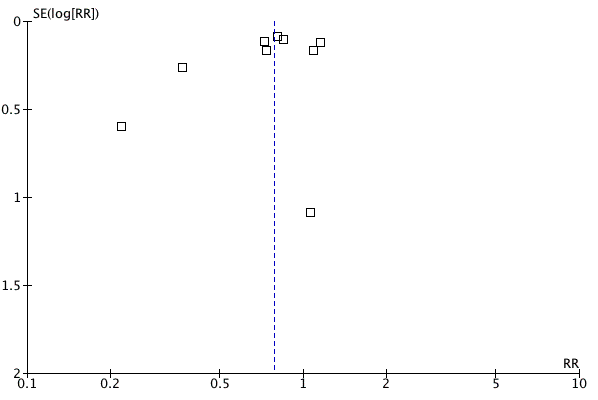
**
